# A novel classification system for evolutionary aging theories

**DOI:** 10.3389/fgene.2013.00025

**Published:** 2013-03-06

**Authors:** Lucas S. Trindade, Toshiro Aigaki, Alexandre A. Peixoto, Alex Balduino, Ivana B. Mânica da Cruz, Jonathan G. Heddle

**Affiliations:** ^1^Heddle Initiative Research Unit, Advanced Science InstituteWako, Saitama, Japan; ^2^Department of Investigative Pathology, Graduate School of Biomedical Sciences, Nagasaki UniversityNagasaki, Japan; ^3^Department of Biological Sciences, Tokyo Metropolitan UniversityHachioji, Tokyo, Japan; ^4^Laboratório de Biologia Molecular de Insetos, Instituto Oswaldo Cruz, FIOCRUZRio de Janeiro, Brazil; ^5^Development and Technology Research Center, Universidade Veiga de AlmeidaRio de Janeiro, Brazil; ^6^Departamento de Morfologia, Centro de Ciências da Saúde, Universidade Federal de Santa MariaSanta Maria, Brazil

**Keywords:** senemorphism, caloric restriction, longevity, altruism, senescence, evolution

## Abstract

Theories of lifespan evolution are a source of confusion amongst aging researchers. After a century of aging research the dispute over whether the aging process is active or passive persists and a comprehensive and universally accepted theoretical model remains elusive. Evolutionary aging theories primarily dispute whether the aging process is exclusively adapted to favor the kin or exclusively non-adapted to favor the individual. Interestingly, contradictory data and theories supporting both exclusively programmed and exclusively non-programmed theories continue to grow. However, this is a false dichotomy; natural selection favors traits resulting in efficient reproduction whether they benefit the individual or the kin. Thus, to understand the evolution of aging, first we must understand the environment-dependent balance between the advantages and disadvantages of extended lifespan in the process of spreading genes. As described by distinct theories, different niches and environmental conditions confer on extended lifespan a range of fitness values varying from highly beneficial to highly detrimental. Here, we considered the range of fitness values for extended lifespan and develop a fitness-based framework for categorizing existing theories. We show that all theories can be classified into four basic types: secondary (beneficial), maladaptive (neutral), assisted death (detrimental), and senemorphic aging (varying between beneficial to detrimental). We anticipate that this classification system will assist with understanding and interpreting aging/death by providing a way of considering theories as members of one of these classes rather than consideration of their individual details.

## INTRODUCTION

**FIGURE 1:**
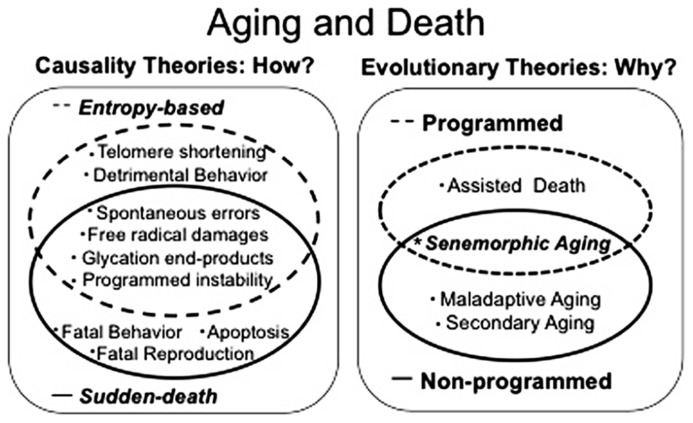
**Aging and death theories can be classified into two groups: (1) causality theories which address questions of how aging and death occur and can be subdivided into entropy-based processes and “sudden death.”** (2) Evolutionary theories which try to explain why species age and die in the way they do. They consist of programmed aging, non-programmed aging and senemorphic aging which is a special case where parallel evolution of “senemorphisms” (independent aging phenotypes encoded by the genome) are related to both a genetic profile to accelerate aging and a genetic profile to maximize lifespan.

After a century of aging research, the dispute over whether the aging process is active or passive persists and a comprehensive and universally accepted theoretical model remains elusive (Jin, 2010). Contradictory data and theories supporting both exclusively programmed and exclusively non-programmed theories continue to grow (Jin, 2010; Mitteldorf, 2010c; Goldsmith, 2011; Martins, 2011). The idea that aging must be either active or passive is fundamentally incorrect because it is surely the case that aging could in principle be active in some species and passive in others. Moreover, some, or possibly all species could have evolved plasticity of lifespan within both programmed and non-programmed aging phenotypes in order to cope with environmental changes; occasionally favoring the kin, occasionally favoring the individual. Clearly different gerontologists have different points of view, and to understand the evolution of aging/lifespan, all data, theories and arguments must be considered and reconciled. To do this, all hypotheses must be classified into a small number of well-understood categories. Here, we offer a fitness-based framework for categorizing existing evolutionary aging theories. Firstly we describe causality theories of death, which are concerned simply with the process of dying (**Figure 1**). They are subdivided into “entropy-based” and “sudden death” mechanisms. Secondly we characterize evolutionary theories of aging, which are concerned with both the selective pressures and the evolutionary processes that could inhibit the evolution of longer lifespan (**Figure 1**). Evolutionary theories consist of “maladaptive aging,” “secondary aging,” “assisted death,” and “senemorphic aging.” This approach should reveal common themes that will prove helpful to researchers. Below, we explain this system of classifying aging/death theories in detail.

## CAUSALITY THEORIES OF DEATH

We make the important distinction between causes of death theories and evolutionary theories of aging (**Figure 1**). Causality theories are solely concerned with the main cause of intrinsic death. Consistently, it has been shown that the proximal causes of aging and death differ depending on species and environmental conditions (see for example: Andrade, 1996; Demetrius, 2005; Rattan, 2006; Greer and Brunet, 2011). We divide these causes into two broad groups: (a) entropy-based: when death follows a relatively long period of degeneration (senescence); (b) sudden-death: when death follows a relatively short period of degeneration or is an almost instantaneous process.

### ENTROPY-BASED THEORIES

Senescence is clearly a deteriorative process featuring increasing disorder. In the course of senescence, intrinsic death ultimately occurs as a result of physical deterioration due to this increasing disorder, e.g., from the accumulation of molecular-level damage. In these situations it is reasonable to say that senescence is the cause of death. Entropy-based theories of aging have advanced extraordinarily in the past three decades, revealing possible causes of aging and death for most species and include: spontaneous errors (e.g., DNA mutations, protein misfolding), free radical damage, advanced glycation end-products, gerontogenes, etc. (Rattan, 2006). More recently it was proposed that a combination of factors rather than a single mechanism is responsible for age-related death (Rattan, 2006). This is an important area of research as an understanding of the processes involved may lead to the design of treatments to inhibit or reverse age-related diseases.

### SUDDEN DEATH

Entropy-based death applies when senescing individuals gradually deteriorate until a tipping point is reached. Sudden death on the other hand is death which occurs in non-senescent individuals over a short time scale or even instantaneously. The classic examples include: (1) fatal reproduction: semelparous species that die rapidly after reproduction (Robertson, 1961; Wodinsky, 1977; Bradley, 2003) or males of some social insect species, which expel their penis in order to enhance fecundity; bringing together the internal organs and automatically killing the animal (Gary and Marston, 1971); (2) cannibalism: in some cases, this is thought to be a result of sexual competition such as in the golden orb-web spider (Schneider et al., 2001). In other cases it is thought to be an important form of death in order to recycle energy in several species (Andrade, 1996; Foellmer and Fairbairn, 2003; Prenter et al., 2006); (3) kin protection: as seen in the female honey bees upon stinging: where the stinger and part of the abdomen remain in the skin of the potential aggressor and releases pheromones to attract more bees (Hunt et al., 2003); (4) apoptosis: proposed as a population survival strategy in unicellular organisms (Lane, 2008).

## CAUSALITY VERSUS EVOLUTIONARY AGING THEORIES

The existence of senescence in no way suggests that biological systems cannot act as islands of reverse entropy to avoid death indefinitely. Indeed the order inherent in living systems is one of their defining features. The Second Law of Thermodynamics asserts that closed systems will become disordered. However, living organisms are open self-organizing systems and thus in principle potentially able to maintain a high level of order. In brief, life exists by using energy to maintain order in the face of entropic pressure (Mitteldorf, 2010a). Some individuals can carry out this process for hundreds of years or more (Medawar, 1952; Abele et al., 2008). Causality theories of aging consider the immediate causes of aging and death. It is self-evident that entropy increases as individuals senesce while enough order is maintained to avoid death. It is unclear why living systems do not continue maintaining order to the same level of stringency indefinitely: we are left searching for evolutionary explanations to understand why at some point in time body maintenance decreases; and why this point is distinct in different species.

The central idea for understanding the evolution of aging/lifespan is straightforward: natural selection favors traits related with efficient reproduction whether they benefit the individual or the kin and much evidence has accumulated in support of this idea (for example: Sundström et al., 1996; Bourke, 2011). As predicted mathematically by Hamilton (1964), interesting recent data using model robotic systems also suggests that altruism will always evolve when the benefits to the kin overwhelm the detrimental effects for the individual (Waibel and Keller, 2011). Consequently, the same principle of a balance between individual and kin benefit could allow aging/death to evolve as an adaptation, an idea supported by several authors (see for example: Andrade, 1996; Crespi and Teo, 2002; Mitteldorf, 2010c; Martins, 2011; Fukuyo et al., 2012).

The segregation of causality from evolutionary theories of aging is thus crucial to avoid any “non-sequitur” fallacy. For instance, it is often suggested that senescence, being a degenerative and detrimental process, cannot be adapted by natural selection. This is logically incorrect – altruistic behaviors are by definition detrimental to individuals yet are believed to have evolved by natural selection. Furthermore, several forms of death (e.g., by self-starvation, submissive cannibalism) have been shown to be beneficial for the kin and are supposed to have evolved for this reason (see examples: Andrade, 1996; Larkin and Slaney, 1997). Workers from social species have the same genetic background as queens and yet do not reproduce and have significantly shorter lifespans. Thus, we must not overlook the fact that some of these altruistic adaptations and senemorphism (i.e. the worker-specific age-related phenotype) are even more detrimental than merely senescence itself.

In conclusion: to understand the evolutionary reasons for species-specific lifespan, we must first understand the balance between the advantages and disadvantages of extended lifespan in the process of spreading genes. This balance is likely to be niche and environment-dependent and thus cannot be understood by metabolism and physiology alone.

## EVOLUTIONARY AGING THEORIES

A central concern of evolutionary aging theories is to track down the population genetic processes restricting the evolution of lifespan. In other words, why species have the lifespan they have rather than a longer or shorter one. As discussed above, whether faster or slower aging will evolve depends on whether or not the chance of spreading genes is increased and so can only be understood by consideration of life-history and environmental conditions. From an evolutionary perspective, every characteristic can be classified for its fitness value, which means a specific trait can be considered neutral, beneficial, or detrimental for the individual or kin under specific environmental conditions. We suggest that the central conflict among evolutionary aging theories is that each theory only attributes one fitness value for longevity (e.g., extended lifespan being exclusively beneficial, neutral, or detrimental). The apparent conflict arises from the fact that each theory describes distinct scenarios that apply different selective pressures on longevity (and reproduction) and so cannot be compared. These different selective pressures determine the type of the evolutionary process affecting the evolution of lifespan potential. Therefore, we classify actual evolutionary aging theories according to a range of fitness values assumed for extended lifespan; highlighting the possible corresponding evolutive processes (**Table 1**). We sub-divide the evolutionary theories of aging into four sub-groups depending on specific selective pressure as follows: (a) maladaptive aging: when fitness associated with extended lifespan is neutral, thus longevity could be arrested or lost by retrogression (mutational load and drift); (b) secondary aging: when fitness associated with extended lifespan is beneficial, yet lifespan potential could be lost by a trade-off (pleiotropy/hitchhiking effect); (c) assisted death: when fitness associated with extended lifespan is significantly detrimental for the kin, thus senescence could evolve as a direct adaption to enhance reproduction; (d) senemorphic aging: when fitness associated with extended lifespan historically varied between beneficial and detrimental depending on changes in environmental conditions, thus parallel senemorphoses (distinct senescence patterns encoded by the genome) could have evolved within the same species (**Table 1**).

**Table 1:** Fitness-based classification system for the evolutionary aging theories.

Fitness value/specific environmental conditions	Theoretical group	Processes inhibiting the evolution of lifespan	Theories examples
The fitness associated with extended lifespan is neutral when the force of natural selection decreases with aging. Longer lifespan cannot evolve and even could be lost by retrogression.	Maladaptive aging	Mutational load + genetic drift (retrogression) Genetic linkage	Mutation accumulation Somatic damage Infectious diseases
The fitness associated with extended lifespan is beneficial but secondary, when it is overwhelmed by another trait. Lifespan could be exchanged for such more beneficial trait.	Secondary aging	Trade-offs (e.g., reproduction) Pleiotropy Hitchhiking effect	Antagonistic Pleiotropy Disposable Soma
The fitness associated with extended lifespan is detrimental when longer lifespan of parents negatively affects the kin fitness. An intrinsic program could have evolve to inhibit extended lifespan (direct adaptation).	Assisted death	Down-regulation of protection Down-regulation of repair Programmed instability Programmed death	Release resources Demographic control Increase variability
The fitness associated with extended lifespan most likely varies between beneficial and detrimental depending on environmental conditions. Distinct adaptations could emerge: one to maximize lifespan and one to inhibit extended lifespan.	Senemorphic aging	*Combination of mechanisms*	Germ-soma conflict Senemorphic aging

*Unifying evolutionary aging theories. The table is based on all possible outcomes for extended lifespan (beneficial, detrimental, neutral, or variable). Column 1 highlights the environmental condition restricting the evolution of longer lifespan. Column 2 shows the respective class of evolutionary aging theory. Column 3 shows the corresponding evolutionary processes restricting the evolution of longer lifespan. Column 4 gives examples of existing theories that fall under these headings.*

According to this view, distinct programs resulting in specific lifespan potential could have evolved to enforce optimal adaptation under different environmental conditions (as seen in social species). To our knowledge this is the first time a classification system has listed all fitness values related with longevity to explain the evolution of lifespan.

## MALADAPTIVE AGING THEORIES

**FIGURE 2:**
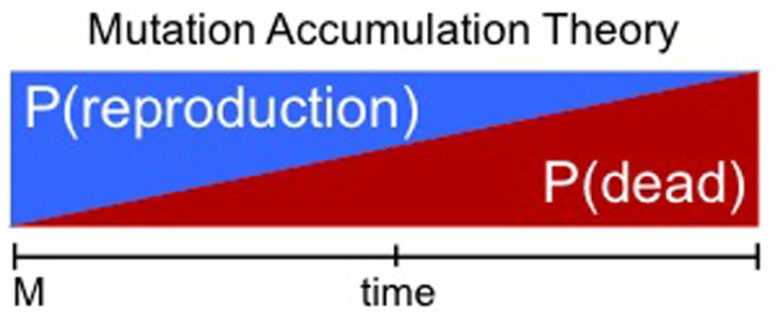
**Maladaptive aging theories**. Illustration of “Mutation Accumulation Theory.” Here the probability of an individual being dead (P(dead), red) increases over time solely due to harsh extrinsic mortality. Consequently the likelihood of successful reproduction (P(reproduction)) at any given time decreases over time (blue). The increasing probability of being dead acts as the main force restricting the selection for longer lifespan. It is reasonable to assume that there is no selective pressure for longevity after a certain threshold at which the likelihood of reproducing is very low. In this case the fitness associated with extended lifespan is neutral. Here death is supposed to occur before senescence has an effect, thus senescence is not necessary to affect the probability of death. M, maturity.

An attractive theory for the existence of death in some species is the declining force of natural selection with age: the lower reproductive efficiency of long-lived individuals will eventually and indirectly lead a species to adapt a shorter lifespan. That the force of natural selection declines with age would be ensured by environmental factors such as: (1) somatic damage (e.g., limb trauma) which could accumulate even in an “immortal” individual (Weismann, 1891); (2) eventual death through extrinsic forces (e.g., predation, accidents; Medawar, 1952); (3) population-wide infectious diseases that cause sterility but not death (Ricklefs, 1998; Kirchner and Roy, 1999). In these environmental conditions the evolution of a longer lifespan is inhibited as a consequence of harsh extrinsic forces (which cause pro-longevity mutations to be ineffective). A well-accepted example of this class of theory is the mutation accumulation theory of Medawar (1952). In this theory, once strong extrinsic mortality imposes a limit on lifespan, then lifespan is supposed to be arrested or even lost by retrogression (mutational load and drift; **Figure 2**). If true, it may be thought that conditions of low extrinsic mortality would lead to evolution of a longer lifespan, independent of an individual’s rate of reproduction. Interestingly, evidence that this is possible can be found in nature and also was demonstrated in laboratory conditions (Rose, 1984; Keller and Genoud, 1997; Møller, 2006). In actual fact, it is now appreciated that changes in longevity via changes in mortality can only occur if the extrinsic mortality is not only strong but also age-dependent (Caswell, 2007). However, in reality as discussed above it is quite reasonable that older individuals could be more vulnerable to extrinsic challenges due to inevitable accumulation of damage over time. In summary, in a maladaptive aging theory, lifespan could in principle always be lengthened, if not for the assumption that extrinsic forces are always extremely harsh. The robust impact of extrinsic forces would invariably impose a longevity maladaptation.

### MALADAPTIVE AGING CANNOT BE UNIVERSAL

According to the predictions of maladaptive aging theories a species lifespan depends upon lifespan being limited by random and harsh extrinsic mortality, which assures the probability of reproduction decreases with age (**Figure 2**). In such conditions, individuals would not have the chance to senesce and thus further increases of lifespan potential would be irrelevant (neutral). However, as demonstrated by Caswell (2007) and mentioned above, extrinsic mortality *per se* could not have an effect on lifespan; effects are only seen if the mortality is age-specific. In support of this, there is strong evidence that slight decreases in fitness due to senescence (an intrinsic process) are enough to negatively affect fecundity and survival of older individuals from some species (Ricklefs, 1998, 2008). This means that we cannot generally assume that it is extrinsic mortality alone which restrains the evolution of lifespan in all species. The works of Ricklefs (1998, 2008) suggest not only that senescence can be seen in nature, but also senescence itself decreases fecundity and survival of individuals from several species. If senescence increases vulnerability to extrinsic forces (predation, infection, etc.) then senescence itself determines lifespan (Mitteldorf, 2010c). In addition, lengthening of lifespan could be extremely detrimental for the kin in several situations (see assisted death section below). In this case, senescence could be selected for as an altruistic trait. Furthermore, it seems to be the case that inter-species competition significantly favors reproduction over longevity under several conditions (see next section). In this case a longevity–reproduction trade-off could restrict the evolution of longer lifespan regardless of extrinsic mortality.

In summary, low extrinsic mortality could allow species to evolve longer lifespan but it seems to be only in cases accompanied by a compensatory effect on overall fecundity (see next section). Interestingly, however, senescence itself seems to determine lifespan in the wild, i.e., extrinsic mortality only exerts an effect on lifespan if senescence already exists. This phenomenon cannot be explained by a maladaptive aging theory, which explains senescence as being the result of extrinsic mortality.

## SECONDARY AGING THEORIES

**FIGURE 3:**
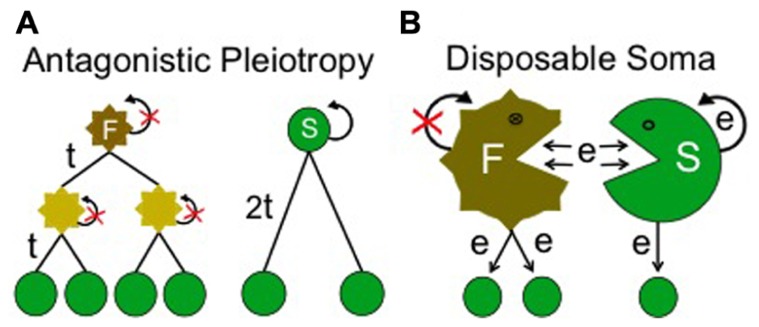
**Secondary aging theories**. This class of evolutionary aging theory assumes that a continuous trade-off restricts selection for longer lifespan. **(A)** Example representing “Antagonistic Pleiotropy” where selection for longevity is assumed to be restricted due to the pressure for faster reproduction. Here a fitter individual (F) is able to produce two offspring with generation time, t. After 2t, individual F has four descendents. In this case, fast reproduction is supposed to cause a side effect on body homeostasis (shown by jagged edges and non-green color) and to minimize regeneration (curly arrow with red cross). A more slowly reproducing individual (S), although more able to regenerate and limit deterioration, is unable to compete under these conditions, as it produces only two descendents in 2t. **(B)** Example representing “Disposable Soma” where selection for longevity is assumed to be restricted due to optimized, efficient utilization of energy (e) for reproduction. Here the fitter individual (F) uses more of the energy consumed to produce offspring rather than regenerate its own body. As a result it deteriorates faster but produces more offspring than an individual (S) which uses more energy for regeneration.

Here, senescence is a result of selection for a trait more useful than maintenance of body fitness after reproduction. In this case, beneficial alleles associated with later life could be exchanged for a more useful trait due to pleiotropy or a hitchhiking effect. By definition, a longevity trade-off can only be justified when the fitness associated with extended lifespan is higher than zero (non-neutral). Antagonistic Pleiotropy, proposed by Williams (1957) is an important example of longevity being restricted by a trade-off for faster reproduction. This theory elegantly suggests how numerous adverse side effects in later life could be maintained by being linked to beneficial effects at younger ages: assuming that faster reproduction is an advantage in the competition among and within species, it will be selected for regardless of accompanying side effects that shorten lifespan (**Figure 3A**; Williams, 1957).

Disposable Soma, proposed by Kirkwood and Holliday (1979) is another important theory of this kind and it is widely appreciated by gerontologists. This theory assumes that both maintenance of soma and reproduction are processes requiring significant amounts of energy. It suggests that organisms in general will adapt to expend resources on optimizing reproduction, trading this off at the expense of soma maintenance. Even if extra energy becomes available, it will be utilized to further optimize reproduction rather than increase lifespan. This is because the theory hypothesizes that optimized reproduction will be always favored over lifespan (**Figure 3B**; Kirkwood and Holliday, 1979). In summary, in a secondary aging theory, the benefits associated with extended lifespan are always considered secondary (non-essential) due to the assumption that a trade-off for faster or increased reproduction would invariably favor the individual.

### SECONDARY AGING CANNOT BE UNIVERSAL

Certainly some niches and conditions favor faster development, growth, and maturation over extended lifespan [e.g., ad libitum (AL) food conditions]. However, secondary aging theories are unlikely to be universally applicable. It must be the case that some environmental conditions favor longevity over fecundity. For example, longevity is positively associated with lower fecundity among species (Holliday, 1995). Indeed, the theory seems not to hold in various examples: Reproduction for females is more costly than for males, yet females of several species live longer than males (Mitteldorf, 2010b). In addition, if reproduction significantly impairs body maintenance in all species, all iteroparous individuals should show a decrease in lifespan with each round of reproduction. This is not the case (Ricklefs and Cadena, 2007). Furthermore, some conditions, i.e., famine, have been shown to in fact favor longevity over reproduction (Holliday, 1989). Most importantly, even given an energetic cost associated with reproduction, there are clearly other environmental conditions in which the selective pressure seems to favor both reproduction and lifespan simultaneously: Solitary insects live for days or weeks, but queens from social species are able to live remarkably longer (reaching almost 30 years) and show significantly higher reproductive capacity (Keller and Genoud, 1997). Naked mole-rats (a species of rodent) are similar in size to mice, but can live up to 30 years and are able to give birth to up to 28 pups at once (Sherman et al., 1999). Finally, Rose (1984), using artificial evolution in *Drosophila melanogaster* demonstrated the selected lines exhibited increased longevity but roughly preserved fecundity through decreased early fecundity and increased later fecundity. After further continued selection, these lines in fact increased early fecundity also (Leroi et al., 1994). The results of Rose prove that it is possible for evolution to increase both lifespan and reproduction at the same time. Yet this does not seem to occur often in nature and when it does it seems to be only in special cases accompanied by a compensatory effect on overall fecundity (e.g., social species where only queens can reproduce). It could be the case that slight increases of longevity are detrimental to kin fitness in nature (e.g., result in parent-offspring conflict, overpopulation, decreased variation).

In conclusion: strong evidence suggests that under several conditions, longevity is in fact favored over reproduction (Holliday, 1995; Mitteldorf, 2010b). However, the most important point to bear in mind here is that it is possible to select increased reproduction and longevity at the same time (Leroi et al., 1994; Keller and Genoud, 1997; Sherman et al., 1999). However, in nature, longer lifespan is always accompanied by a compensatory effect on overall fecundity (either through low numbers of offspring or zero/low potential fecundity of offspring produced). Could even a slight increase in lifespan be detrimental for the spreading of genes?

## ASSISTED DEATH THEORIES

Faster reproduction and genetic variability of a species are crucial for adaptability within a population (Angelo and Van Gilst, 2009). It has been suggested that overpopulation is detrimental to the kin, enforcing suppression of reproduction (Bourke, 2007; Mitteldorf, 2010c; Ronce and Promislow, 2010). Thus, it could be the case that even a small degree of superfluous longevity is enough to cause a detrimental effect on fitness during harsh competition in the wild. Martins (2011), using computational simulations demonstrated that reproduction is crucial for adaptability, while longevity is detrimental. These results raise the distinct possibility that evolution may adapt a genetic pathway to inhibit useless and otherwise harmful increases in longevity. As discussed above, even if senescence is detrimental to the individual, natural selection could still favor faster death if it enhances the fitness of the kin. If one accepts that longevity could be traded-off to enhance individual reproduction (Kirkwood and Holliday, 1979), it also must be acceptable that longevity could be traded-off to enhance kin reproduction (exactly the same effect and in agreement with natural selection). This suggests the possibility of the existence of a “senescence program” to ensure death in order to inhibit or delay the evolution of longer lifespan. Note, however, that such a “program” need not be a direct set of genetic instructions to die: Remembering that rigorous repair and maintenance is constantly required to preserve an organism as an island of negative entropy then we see that the senescence “program” could simply be the adaptation of “master controls” to down-regulate these maintenance processes. In this sense and due to the lack of evidence for a genetic program for death, we can consider senescence as adapted/programmed through selection for mechanisms of decreased protection and repair. In this case the term “assisted death” is more appropriate than “programmed death” in the sense that death is “passively” encoded by the genome. Thus, it would be difficult or impossible to differentiate the molecular mechanisms involved in programmed versus non-programmed senescence.

Acknowledging that evolution could favor reproduction and longevity at the same time (see secondary aging), it is difficult to determine if any down-regulation of body maintenance is a side-effect or an adaptation. For instance, the specific age-related changes during AL conditions clearly are not adapted to optimize body fitness (Trindade et al., 2012). In any case, the significant age-related decrease in body maintenance during AL conditions cannot be uncritically assumed to be a side effect of metabolism and growth. Could for ecological reasons the AL genetic profile be in fact an adaptation?

Several reasons have been hypothesized to favor the adaptation of assisted death: (1) release of resources for offspring; (2) for demographic control; (3) to speed up adaptation (Weismann, 1891; Woolhouse, 1967; Wodinsky, 1977; Kirkwood and Holliday, 1979; Bradley, 2003; Mitteldorf, 2004; Lane, 2008). These ideas are strongly supported by the discovery of genes and mechanisms whose sole role appears to be to decrease lifespan (Kenyon, 2005; Lane, 2008).

Semelparous strategies are the most commonly suggested examples of assisted aging/death. For example, in a range of different phyla, including mollusks, fish, reptiles, and mammals an apparently unnecessary “self-starvation” of parents during and after the breeding season is observed (Woolley, 1966; Wodinsky, 1977; Larkin and Slaney, 1997; Bradley, 2003). These species often live in hash environments where it is plausible to think that abstention from food releases resources for the offspring, increasing their chances of survival. In the ultimate example, in some species the parents’ dead bodies themselves provide, directly or indirectly, a crucial source of food for the young (Andrade, 1996; Watkinson, 2000). Indeed simple mathematical models of semelparity in animals and plants predict that it will be favored when a small number of simple criteria are met such as increased juvenile survivorship and population growth (benefiting the kin; Young, 1981).

Examples supporting the idea of an assisted death adaptation such as mentioned above are generally of the “sudden death” variety where death occurs quickly and it is easy to quantify the benefits to the kin. However, if one accepts this evidence on the basis that parents’ death is beneficial or even crucial for the kin in some niches and environmental conditions, then it may be possible that more gradual death (entropy-based aging) is also a viable means to achieve the same goal. Ultimately, both entropy-based and sudden death could be direct adaptations, particularly if a slight decrease in body maintenance is enough to significantly increase the chance of extrinsic mortality as proposed by Ricklefs (1998, 2008). In summary, in an assisted death theory, lifespan could always be lengthened, if not for the assumption that longer lifespan would invariably cause a detrimental effect on spreading genes. In this case it is postulated that an assisted death program would evolve to benefit the kin.

### ASSISTED DEATH CANNOT BE UNIVERSAL

The benefits of assisted death (programmed aging) have been discussed exhaustively in the literature and briefly above. Nevertheless it seems unlikely that assisted death is universally and irreversibly applicable. In many cases a persistent, “strict” assisted death program would be detrimental for an individual that is unable to reproduce. For example, a starved individual incapable of reproducing, must in fact not die and must survive until food returns and successful reproduction becomes possible (Holliday, 1989). Also a semelparous individual which fails to breed in a particular breeding season must in fact not die and must survive until the subsequent breeding season (Bradley, 2003). Therefore, even if an assisted death program has been shown to significantly favor the kin, in several situations its irreversible activation would be detrimental for the individual and for the kin.

The main challenges for an assisted death theory be accepted are: (1) to propose a genetic pathway leading to death; (2) to suggest how this pathway was maintained during the course of evolution by natural selection. Nevertheless, we do not need to understand how death could evolve and be maintained by natural selection to assume it could be possible. It is still somewhat unclear how sexual reproduction, sociality, and altruism had evolved, but it is clear they did. Faster death of parents by an assisted death program, mainly by self-starvation would likely release food for the offspring, decelerate population growth, increase species genetic variability, and thus the adaptability rate. Thus, some environments may directly favor the adaptation of assisted death. On the other hand, some environments surely favor longevity. This conclusion leads us to a possible unification of aging theories, discussed in the next section.

## SENEMORPHIC AGING THEORIES

Recently, we have highlighted the existence of environment-dependent senemorphic strategies (independent aging patterns encoded by the genome). The evolution of independent genetic pathways to enforce distinct lifespan potentials can be easily identified for example in social species (caste-related senemorphism) where workers and queens have the same genetic background, but show distinct aging patterns modulated by differential gene expression (Trindade et al., 2012). However, the most common senemorphic adaptation among species is the distinct and independent aging patterns of individuals undergoing AL versus caloric restriction (CR) feeding (Trindade et al., 2012). This diet-related “plasticity” in lifespan we termed “diet-related senemorphism.” In brief, there is ample evidence that the response to AL and CR conditions are independent adaptations, as we previously stated: “(1) comparing the two dietary groups, several age-related changes run in the opposite direction over time; (2) switching from an AL to a CR diet clearly reverts (not only delays) several “normal” accumulated changes; (3) major causes of death are as different between both groups as they are between species.” These observations strongly support the idea that independent genetic pathways evolved to modulate distinct lifespan potential during different food conditions. Such an ability to activate a particular genetic program when it is advantageous to do so has been, in the case of social insects, referred to as “parallel evolution of phenotypes” (Rajakumar et al., 2012). In the case of the parallel evolution of aging patterns, we use the term “senemorphic aging” (Trindade et al., 2012). The environment-dependent regulation of lifespan potential offers a good example of how it is of benefit to switch longevity strategies as an adaptation. Surely, the efficient spreading of genes may be favored by either an extended lifespan or a shortened one (**Table 1**). Therefore, here we propose the possibility that the evolution of these distinct genetic pathways are in fact related to the adaptation of both a genetic profile to accelerate aging (altruism, AL, assisted death) and a genetic profile to maximize lifespan (selfishness, CR, maladaptive, and secondary aging). Such distinct aging patterns allow individuals to cope with environmental changes by optimizing indirectly both short and long-term reproduction (**Table 1**).

In summary, senemorphic theory suggests the possibility that the observed diet-related “plasticity” of lifespan potential is a result of direct adaptation for different environmental conditions with long-term activation or deactivation of energy sensing pathways selecting a different downstream cascade. In this case, AL cascade activation could be related to an altruistic program for faster death while the CR cascade could be related to an “individual selection” program to increase lifespan.

### DISCUSSION OF SENEMORPHIC AGING THEORIES

Some authors strongly support the evolution of universal active aging, while others strongly support universal passive aging. The genetic mechanisms associated with diet-related lifespan potential have been evolving conservatively since unicellular life (Flatt and Schmidt, 2009). Therefore, the evolution of pluricellularity (including sexual reproduction) only appeared after the adaptation of diet-related senemorphism (energy sense pathways controlling antagonistically longevity and reproduction). Consequently, a universal evolutionary theory of aging must consider the drastic fluctuations of food availability that species have experienced since LUCA (last universal common ancestor). Since different food conditions determine the fitness associated with extended lifespan, the hypothesis that the AL genetic profile is in fact a direct pro-senescence adaptation and the CR genetic profile is related to the adaptation for an optimized extended homeostasis gives the best explanation for the evolution of aging/lifespan. In this section we offered a general aging theory in which the evolution of appropriate response to available energy results in a strategy that is able to explain all current ideas and evidence discussed above.

In this work, we have not attempted to explain how senemorphic aging evolved, merely to show that it could have been advantageous to have done so. Senemorphic aging offers a useful perspective as it potentially unifies evolutionary aging theories enabling a new perspective in gerontology (**Table 1**).

## CONCLUSION

Currently, evolutionary aging theories are unable to explain convincingly how and why species have a limited lifespan. Each aging hypothesis has significant flaws that we have discussed briefly and which were elegantly described by Mitteldorf (2010a,b,c). It remains to be seen if a single hypothesis can be developed which is able to unify all of these often contradictory ideas into a single aging theory. The major challenge of a universal evolutionary aging theory is to reconcile the possible existence of trade-offs (pleiotropy or hitchhiking effect), retrogression (mutational load and drift) and direct adaptation (“program”). From this perspective, we have described a universal classification system for aging theories (**Table 1**). Our novel framework based on discriminatory selective pressures categorizes aging/death theories as secondary aging, maladaptive aging, assisted death, or senemorphic aging. Considering an individual theory at the level of the category to which it belongs will assist in judging its merits and should help to bring clarity to the field.

Each category is supported by theoretical and experimental data and are not necessarily mutually exclusive: while increase of biological entropy is an important contribution to a limit for lifespans, maladaptive and secondary aging theories suggest that extrinsic forces restrict the evolution of lifespan (through extrinsic mortality and inter-species competition, respectively). Thus, in beneficial environmental conditions lifespan could be lengthened. Is it always beneficial to increase lifespan if the environmental conditions allow it? Increases of longevity are related to decreases of fecundity (Holliday, 1995). Therefore, an altruistic behavior as described by assisted death could accelerate the turnover of generations maintaining the reproductive rate and at the same time releasing resources for offspring. This would clearly be beneficial for the spread of genes leading to the adaptation of assisted death being favored by some environments. In contrast, some environmental conditions (e.g., starvation, non-breeding semelparous animals) surely favor longevity as far as possible.

This conclusion leads us to a possible unification of aging theories: the existence of environment-dependent lifespan programs encoded by the genome could account for both active and passive aging programs (Trindade et al., 2012). Senemorphic aging could be an adaptation for variable environmental conditions, sometimes favoring the kin (e.g., as an assisted death program under AL conditions and in breeding semelparous individuals), sometimes favoring the individual (e.g., as non-programmed aging under CR conditions and in non-breeding semelparous individuals; **Table 1**).

## Conflict of Interest Statement

The authors declare that the research was conducted in the absence of any commercial or financial relationships that could be construed as a potential conflict of interest.
